# The Durability of Vaccine Efficacy against Ocular HSV-1 Infection Using ICP0 Mutants 0∆NLS and 0∆RING Is Lost over Time

**DOI:** 10.3390/pathogens10111470

**Published:** 2021-11-12

**Authors:** Daniel J. J. Carr, Amanda Berube, Edward Gershburg

**Affiliations:** 1 Department of Ophthalmology, Microbiology and Immunology, University of Oklahoma Health Sciences Center, Oklahoma City, OK 73104, USA; 2 Department of Ophthalmology, University of Oklahoma Health Sciences Center, Oklahoma City, OK 73104, USA; Amanda-berube@ouhsc.edu; 3 Rational Vaccines, Inc., Cambridge, MA 02139, USA; ed.gershburg@rationalvaccines.com

**Keywords:** herpes simplex virus type 1, cornea, vaccine, neovascularization, visual acuity

## Abstract

Vaccines to viral pathogens in experimental animal models are often deemed successful if immunization enhances resistance of the host to virus challenge as measured by cumulative survival, reduction in virus replication and spread and/or lessen or eliminate overt tissue pathology. Furthermore, the duration of the protective response against challenge is another important consideration that drives a vaccination regimen. In the current study, we assessed the durability of two related vaccines, 0∆NLS and 0∆RING, against ocular herpes simplex virus type 1 (HSV-1) challenge in mice thirty days (short-term) and one year (long-term) following the vaccine boost. The short-term vaccine efficacy study found the 0∆RING vaccine to be nearly equivalent to the 0∆NLS vaccine in comparison to vehicle-vaccinated mice in terms of controlling virus replication and preserving the visual axis. By comparison, the long-term assessment of the two vaccines found notable differences and less efficacy overall as noted below. Specifically, the results show that in comparison to vehicle-vaccinated mice, the 0∆NLS and 0∆RING vaccinated groups were more resistant in terms of survival and virus shedding following ocular challenge. Moreover, 0∆NLS vaccinated mice also possessed significantly less infectious virus in the peripheral and central nervous systems but not the cornea compared to mice vaccinated with vehicle or 0∆RING which had similar levels. However, all vaccinated groups showed similar levels of blood and lymphatic vessel genesis into the central cornea 30 days post infection. Likewise, corneal opacity was also similar among all groups of vaccinated mice following infection. Functionally, the blink response and visual acuity were 25–50% lower in vaccinated mice 30 days post infection compared to measurements taken prior to infection. The results demonstrate a dichotomy between resistance to infection and functional performance of the visual axis that collectively show an overall loss in vaccine efficacy long-term in comparison to short-term studies in a conventional prime-boost protocol.

## 1. Introduction

Herpes simplex virus 1 (HSV-1) is a large double-stranded DNA virus that consists of approximately 152 kilobase of DNA with over 80 genes encoded by the virus [1]. As HSV-1 has co-evolved with the human host, a number of countermeasures to the host’s innate and adaptive immune response are encoded by the virus that allows it to be one of the more successful human virus pathogens with greater than 3.5 billion individuals infected [2,3]. Owing to the success of HSV-1 includes the neurotropic nature of the virus which allows it to invade and establish latency in neurons that reside in the peripheral and central nervous system of the naive host as well as reactivate clinically or sub-clinically in seemingly immunocompetent individuals [4,5,6]. Of note, it has been found that HSV-1 seropositive individuals that do not experience recurrent herpetic disease retain polyfunctional effector memory CD8^+^ T cells to select HSV-1 polypeptides compared to symptomatic individuals and such polypeptides used in cocktail vaccines have been shown to be efficacious in protecting mice against ocular HSV-1 challenge [7,8,9,10]. Although these vaccines tend to be directed primarily at driving a CD8^+^ T cell response, such studies have identified a T lymphocyte-based explanation for individuals that experience HSV-1 reactivation and emphasize the contribution of CD8^+^ T cells in HSV-1 surveillance in latent-infected individuals as suggested in mouse studies [11,12].

The general basis of vaccination to infectious pathogens is to protect the patient from subsequent exposure to the insulting microbe by enlisting the activation of B and T lymphocytes specific for the agent. Ideally, vaccine-induced lymphocyte activation leads to the generation of memory B and T cells that can rapidly respond to exposure or re-exposure to the microbial insult. Consequently, the efficacy of a vaccine is defined by the magnitude and duration of a protective immune response [13]. The generation of a successful vaccine against HSV-1 is a challenge due to the virus-encoded immune countermeasures directed at the innate and adaptive immune systems, the establishment of latency in neurons that allows the virus to “hide” from probing lymphocytes and circulating antibodies and the subclinical reactivation nature of the virus in a large reservoir of infected individuals. Experimental vaccines against HSV-1 consists of heat-killed virus, live-attenuated virus, virus-encoded protein subunits (typically glycoproteins) and subunit cocktails [14,15,16,17,18,19,20,21,22,23,24]. These vaccines have been deemed successful by the reporting investigative team based on providing sterile immunity, the induction of a robust immune (B and/or T cell) response and in some instances, reduction of tissue inflammation. While some of these vaccines may have value in preventing or reducing the severity or incidence of herpes labialis, a common manifestation of HSV-1 infection [25], the efficacy of these experimental vaccines in protecting the integrity and function of sensitive anatomical regions including the eye have not been addressed or have done so using qualitative means that do not consider functional consequences of the visual axis in parameters under analysis.

We have previously described the successful use of a live-attenuated virus as a vaccine against ocular HSV-1 challenge with the genesis of a robust immune response and preservation of corneal integrity and visual function [26,27]. The efficacy of the vaccine was found to be dose-dependent and did not require the presence of the neonatal Fc receptor [28,29]. The basis of the attenuated virus used as a vaccine stems from earlier work identifying the immediate early gene, infected cell protein (ICP) 0 as a crucial component of the virus in resistance against type I interferon (IFN) pathway activation [30,31,32,33]. The creation of the ICP0 mutant in which the nuclear location signal (NLS) is disrupted led to a virus (denoted HSV-1 0∆NLS) that can infect and replicate in cells but at an appreciable loss compared to wild type (WT) parental virus associated with high sensitivity to type I IFN [26]. While HSV-1 0∆NLS was found to be a highly efficacious prophylactic vaccine against ocular HSV-1 challenge, the duration of protection of the vaccine was not evaluated. Another HSV-1 ICP0 mutant, 0∆RING derived from the deletion of codons 105–229 of the ICP0 gene and, therefore, negate of E3 ligase activity [34], was used as a comparative ICP0 mutant vaccine to the 0∆NLS vaccine. The current study was undertaken to determine if vaccinated mice retained resistance against subsequent challenge one year following the vaccine boost relative to a short-term efficacy study in which mice were challenged with HSV-1 30 days post vaccine boost.

## 2. Results

### 2.1. A Comparison of HSV-1 ICP0 Mutants 0∆NLS and 0∆RING to Parental (WT) GFP105 Resistance to IFN-β In Vitro

**Figure 1:**
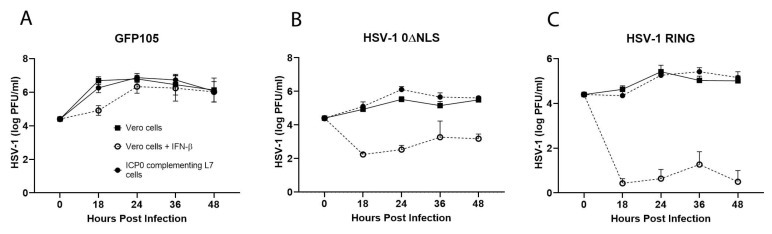
HSV-1 replication efficiency of wild type and ICP0 null mutant viruses. Titers of (**A**) HSV-1 WT GFP105, (**B**) HSV-1 0∆NLS and (**C**) HSV-1 0∆RING at times after inoculating Vero cells, IFN-β-treated Vero cells, or ICP0^+^ L7 cells with 0.1 PFU per cell. At each time point, cultures were collected and frozen at –20 °C until titers were determined by plaque assay. The mean + SEM (*n* = 6–9 samples/time point) is plotted as a function versus IFN-β (200 U IFN-β/culture)-treated Vero cells. Each graph is a summary of the results for each virus from 2–3 experiments.

Previous studies have reported HSV-1 ICP0 null viruses do not efficiently replicate in cells that have been exposed to type I IFN [30,35]. To determine if HSV-1 ICP0 mutants 0∆NLS and 0∆RING were equally sensitive to type I IFN, the parental WT GFP105 and HSV-1 ICP0 mutant viruses were analyzed for their growth kinetics in cell monolayers of Vero cells, Vero cells treated with IFN-β, or ICP0-complementing L7 cells. In parental WT GFP105 infected cells, IFN-β pretreatment of Vero cells modestly reduced virus replication at the 18 hr time point 60-fold but latter time points from 24–48 h pi, IFN-β pretreatment had a negligible effect (Figure 1A). By comparison, the ICP0 mutants 0∆NLS (Figure 1B) and 0∆RING (Figure 1C) were dramatically affected by IFN-β pretreatment with losses ranging from 76- to over 60,000-fold in replication over 48 h pi. The ICP0 0∆RING mutant was the most impacted by IFN-β consistent with a previous study evaluating HSV-2 ICP0 mutant virus replication in Vero cells with and without IFN-β pretreatment [36]. Thus, like the HSV-2 ICP0 mutant counterparts, HSV-1 ICP0 mutants 0∆NLS and 0∆RING are exquisitely sensitive to the anti-viral effect of type I IFN.

### 2.2. The ICP0 Mutant 0∆RING Shows Similar Degrees of Efficacy Compared to the ICP0 Mutant 0∆NLS as A Prophylactic Vaccine against HSV-1 in Mice Challenged 30 Days Post Boost

**Figure 2:**
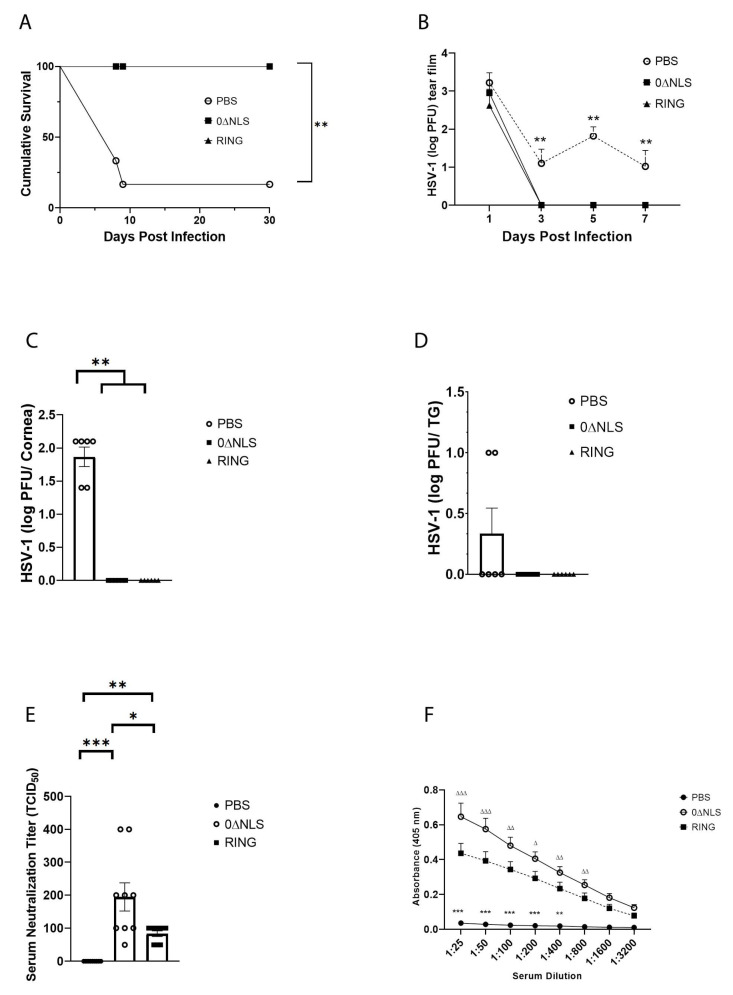
HSV-1 0∆RING ICP0 mutant shows similar efficacy to HSV-1 ICP0 mutant 0∆NLS as a prophylactic vaccine against ocular virus challenge in mice over a short-term duration. C57BL/6 WT male and female mice (*n* = 6–12/group/measurement) were vaccinated with HSV-1 0∆NLS (1 × 10^5^ PFU), HSV-1 0∆RING (1 × 10^5^ PFU) or vehicle (PBS) and subsequently ocularly challenged with HSV-1 McKrae (1 × 10^4^ PFU/cornea) 30 days post vaccine boost. (**A**) Mice were monitored for survival out to day 30 post infection (pi). The results are the summary of two independent experiments; ** *p* < 0.01 comparing the HSV-1 0∆NLS and 0∆RING vaccinated groups to the vehicle-vaccinated mice (6 mice/group) as determined by Mantel Cox test day 9-day 30 pi. (**B**) Virus shedding in tear film of vaccinated mice over time pi. The results depict mean + SEM, *n* = 6–7 mice/group at each time point. ** *p* < 0.01, comparing vehicle-vaccinated mice to 0∆RING- and 0∆NLS-vaccinated animals at days 3–7 pi as determined by ANOVA and Scheffé multiple comparison test. (**C**,**D**) Virus titers were determined by plaque assay at day 7 pi in the (**C**) cornea and (**D**) TG of the vaccinated animals (6 mice/group). ** *p* < 0.01 comparing the indicated groups as determined by ANOVA and Scheffé multiple comparison test. Sera was collected from vaccinated mice 30 days post boost and evaluated for (**E**) virus neutralization titers and (**F**) reactivity to HSV-1 antigen. The results are expressed as mean + SEM (*n* = 6–12/group), *** *p* < 0.001, ** *p*< 0.01, * *p*< 0.05 comparing the indicated groups in (**E**) and *** *p* < 0.001, ** *p* < 0.01 comparing the PBS- to 0∆NLS and 0∆RING-vaccinated groups in (**F**). ^∆∆∆^ *p* < 0.001, ^∆∆^ *p* < 0.01 and ^∆^ *p* < 0.05 comparing the 0∆NLS- to RING-vaccinated groups in (**F**) all determined by two ANOVA and Tukey’s multiple comparison tests.

Initially, we sought to determine if the HSV-1 ICP0 mutant RING used as a prophylactic vaccine protected mice to the same degree as the related HSV-1 ICP0 mutant, 0∆NLS previously reported to show significant efficacy against subsequent ocular virus challenge [26,27]. Similar to 0∆NLS-vaccinated mice, 0∆RING-immunized animals did not succumb to HSV-1 infection when challenged 30 days post-boost whereas greater than 80% of phosphate-buffered saline (PBS, vehicle)-vaccinated animals died following primary infection by day 9 pi (Figure 2A). Consistent with these results 0∆RING-vaccinated mice shed significantly less virus during acute infection (Figure 2B) and maintained no measurable infectious virus in the cornea (Figure 2C) or TG (Figure 2D) at day 7 pi similar to 0∆NLS-vaccinated animals in comparison to PBS-immunized controls. However, there was a noticeable difference in neutralizing antibody titers (Figure 2E) and anti-HSV-1 antigen reactivity (Figure 2F) comparing 0∆RING to 0∆NLS-vaccinated mice. Whereas both ICP0 mutant vaccinated groups displayed elevated neutralizing antibody titers and HSV-1 antigen reactivity compared to the PBS-immunized mice, 0∆NLS-vaccinated animals sera possessed significantly greater antibody neutralizing activity (Figure 2E) and HSV-1 antigen reactivity (Figure 2F) compared to the RING-immunized animals.

**Figure 3:**
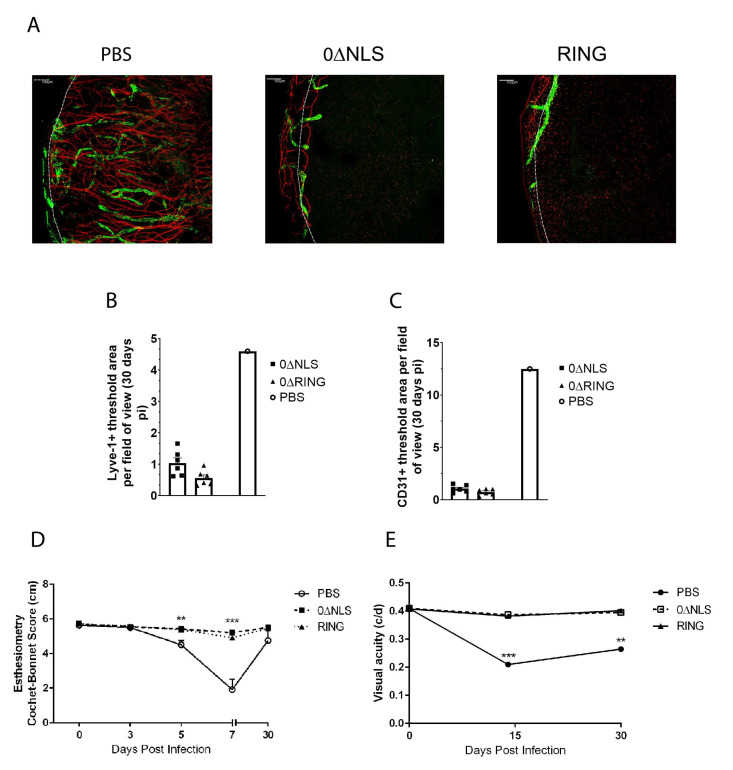
HSV-1 ICP0 mutant 0∆RING vaccine preserves the visual axis of mice following ocular HSV-1 challenge 30 days post vaccine boost. C57BL/6 WT male and female mice (*n* = 6/group) were vaccinated with HSV-1 0∆NLS (1 × 10^5^ PFU), HSV-1 0∆RING (1 × 10^5^ PFU) or vehicle (PBS) and subsequently ocularly challenged with HSV-1 McKrae (1 × 10^4^ PFU/cornea) 30 days post vaccine boost. (**A**–**C**) At 30 days post infection (pi), mice were exsanguinated and the corneas were processed and assessed for neovascularization. Representative figures for each vaccinated group are shown in panel A. The summary of the area of the cornea occupied by lymphatic (LYVE-1^+^ green) and blood (CD31^+^ red) vessels is included in panel B and C respectively. (**D**) Vaccinated mice were assessed for the blink response using a Cochet-Bonnet esthesiometer prior to and at the indicated times pi. The results are reported as the mean length (cm) to yield a blink response + SEM for each group. (**E**) Visual acuity scores were determined by using optomotry prior to and following HSV-1 infection of vaccinated mice. The results are reported as the mean for each group at each time point. *** *p* < 0.001, ** *p* < 0.01 comparing the PBS- to the 0∆RING and 0∆NLS vaccinated groups as determined by ANOVA and Tukey’s post-hoc multiple comparison test for results analyzed in panels (**D**,**E**).

Next, vaccinated mice were evaluated for cornea pathology by measuring virus-induced corneal neovascularization. Similar to 0∆NLS-vaccinated animals, 0∆RING-immunized mice showed no appreciable corneal neovascularization (blood and lymphatic vessel genesis) at day 30 pi compared to PBS-vaccinated animals which displayed robust blood and lymphatic vessel growth into the central cornea (Figure 3A–C). From a functional standpoint, RING-immunized mice maintained a blink response (Figure 3D) and visual acuity (Figure 3E) similar to 0∆NLS-vaccinated animals in comparison to PBS-vaccinated mice that displayed a temporal or sustained loss in mechanosensory function (Figure 3D) and visual acuity (Figure 3E) respectively. Collectively, these results demonstrate the ICP0 mutant 0∆RING has a nearly identical efficacious profile to that of the ICP0 mutant 0∆NLS when used as a prophylactic vaccine against ocular HSV-1 infection challenged 30 days post vaccine boost. Whether the ICP0 mutants provide equivalent protection long-term was investigated next.

### 2.3. The HSV-1 0∆NLS Vaccine Displays Superior Efficacy Compared to The HSV-1 0∆RING Vaccine as Determined by Virus Replication and Weight Loss Results following Ocular HSV-1 Challenge in Long-Term Study

**Figure 4:**
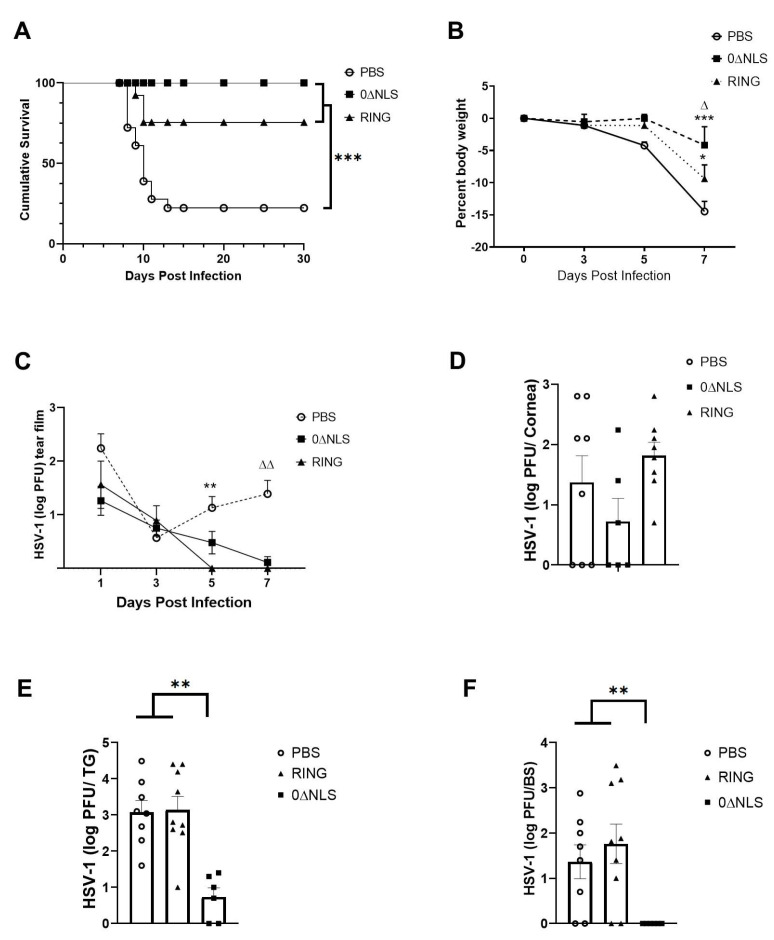
HSV-1 ICP0 mutant viruses 0∆NLS and 0∆RING used as prophylactic vaccines show durability in protection against HSV-1-mediated mortality long-term. C57BL/6 WT and Rosa male and female mice (*n* = 6–18/group/measurement) were vaccinated with HSV-1 0∆NLS (1 × 10^5^ PFU), HSV-1 0∆RING (1 × 10^5^ PFU) or vehicle (PBS) and subsequently ocularly challenged with HSV-1 McKrae (1 × 10^4^ PFU/cornea) 365 days post boost. (**A**) Mice were monitored for survival out to day 30 post infection (pi). The results are the summary of five independent experiments; *** *p* < 0.001 comparing the HSV-1 0∆NLS and 0∆RING vaccinated groups to the vehicle-vaccinated mice (15–18 mice/group) as determined by Mantel Cox test day 12-day 30 pi. (**B**) Mice (*n* = 5 mice/group) were evaluated for % body weight loss over the first 7 days pi. The results depict mean + SEM, *** *p* < 0.001, * *p* < 0.05 comparing 0∆RING- and 0∆NLS-vaccinated mice to vehicle-vaccinated animals respectively at day 7 pi. ^∆^ *p* < 0.05 comparing the 0∆NLS vaccinated WT to the 0∆RING-vaccinated mice as determined by two-way ANOVA and Tukey’s post hoc t-test at day 7 pi. (**C**) Virus shedding in tear film of vaccinated mice over time pi. The results depict mean + SEM, *n* = 9–16/group at each time point. ** *p* < 0.01, comparing 0∆RING-vaccinated mice to vehicle-vaccinated controls at day 5 pi. ^∆∆^ *p* < 0.01 comparing vehicle-vaccinated mice to 0∆RING- and 0∆NLS-vaccinated animals at day 7 pi as determined by ANOVA and Scheffé multiple comparison test. (**D**–**F**) Virus titers were determined by plaque assay at day 7 pi in the (**D**) cornea, (**E**) trigeminal ganglia (TG) and (**F**) brain stem (BS) of the vaccinated animals (6–9 mice/group). ** *p* < 0.01 comparing the indicated groups as determined by ANOVA and Scheffé multiple comparison test.

To calibrate the long-term durability of the HSV-1 0∆NLS vaccine against subsequent challenge with HSV-1, vaccinated mice were infected one year following the boost using the prime-boost vaccination protocol [26] and monitored over the course of 30 days. In comparison to vehicle (PBS)-vaccinated animals, HSV-1 0∆NLS- and 0∆RING-vaccinated mice did not succumb to primary infection following virus challenge with all (21/21) in the case of the 0∆NLS-vaccinated group or the majority (12/15) in the case of the 0∆RING-vaccinated group surviving acute infection (Figure 4A). By comparison, greater than 74% of PBS-vaccinated mice (14/18) succumbed to infection. These results are consistent with the dramatic drop in weight of PBS-vaccinated mice following challenge over the course of 7 days pi approaching 15% loss in comparison to 0∆RING- (9% loss) and 0∆NLS (4% loss)-vaccinated groups (Figure 4B).

To further define the level of protection in the HSV-1 ICP0 mutant-vaccinated mice, virus shedding, replication and spread were evaluated in infected mice. In comparison to PBS-vaccinated mice, 0∆RING-vaccinated mice possessed no detectable infectious virus in the tear film by day 5 pi whereas the majority (15/16) of 0∆NLS vaccinated mice did not effectively eliminate virus until day 7 pi (Figure 4C). In contrast, the majority (12/15) of PBS-vaccinated mice retained infectious virus shedding as detected in the tear film at day 7 pi which were significantly elevated compared to the 0∆RING- or 0∆NLS vaccine mice (Figure 4C). These results, however, did not reflect infectious virus levels found in the cornea tissue at day 7 pi. Specifically, there was no significant difference in viral loads obtained from the cornea comparing PBS- to 0∆RING- and 0∆NLS- vaccinated animals (Figure 4D). Nevertheless, 0∆NLS-vaccinated mice possessed significantly less virus in the TG and BS compared to PBS- and 0∆RING-vaccinated groups which had nearly equivalent levels of recoverable infectious virus (Figure 4E,F). Collectively, the results suggest the 0∆NLS-vaccinated mice show the greatest resistance to virus challenge with significant integrity in the anti-viral efficacy intact with the exception of cornea virus loads that were found to be similar between all groups of vaccinated mice in this long-term study.

### 2.4. Anti-HSV-1 Antibody Titers Are Lost over Time following Vaccination with 0∆NLS or 0∆RING

**Figure 5:**
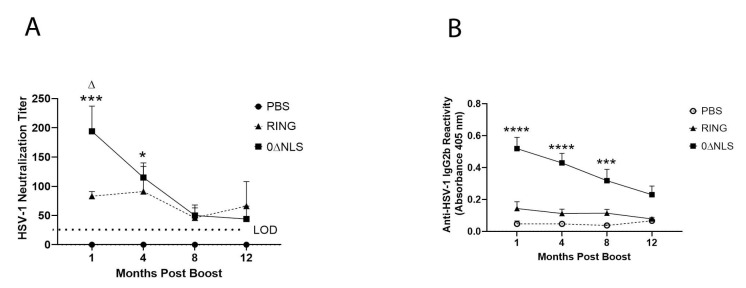
Neutralizing antibody titer in 0∆NLS-vaccinated mice is diminished over time. Mice (18–22 mice/group) were vaccinated as described under materials and methods section with either PBS (vehicle), 0∆RING, or 0∆NLS ICP0 mutants. At 1, 4, 8, and 12 months post-boost, blood was collected from each animal and the sera was evaluated for (**A**) virus neutralization titers and (**B**) reactivity to HSV-1 antigen. Each time point represents the mean + SEM, **** *p* < 0.0001, *** *p* < 0.001, * *p* < 0.05 comparing the 0∆NLS to the PBS-vaccinated group. ^∆^ *p* < 0.05 comparing the 0∆NLS- to 0∆RING-vaccinated groups as determined by two ANOVA and Tukey’s multiple comparison tests. LOD = limit of detection for (**A**).

Previous studies have found antibody neutralizing titers correlate with the efficacy of the HSV-1 0∆NLS vaccine delivered prophylactically 30 days prior to ocular HSV-1 infection [26,27]. Therefore, antibody neutralization titers to HSV-1 were determined from sera obtained from vaccinated mice at times post booster prior to challenge. The results show that at 1 and 4 months post-boost, mice that received the 0∆NLS vaccine possessed a significantly higher neutralization titer compared to the vehicle-immunized group (Figure 5A). Likewise, at 1 month post-boost, 0∆NLS vaccinated mice maintained a significantly elevated neutralization titer against HSV-1 compared to mice immunized with the 0∆RING vaccine (Figure 5A). However, by 8 months through 12 months post-boost, the neutralization antibody titers to HSV-1 were lost in the 0∆NLS- and 0∆RING-vaccinated animals with no meaningful difference in levels compared to the vehicle-vaccinated mice (Figure 5A). Therefore, a prime-boost vaccination regimen using the ICP0 mutants does not maintain a measurable neutralization antibody titer to HSV-1 past 4 months post boost. Similar to the neutralizing antibody titers, reactivity to HSV-1 antigen by antisera from 0∆NLS vaccinated mice was significantly attenuated over time (Figure 5B). As expected, significant reactivity was found 1–8 months post-vaccine boost. However, there was a steady decline in recognition of HSV-1 antigen such that by 12 months post-boost, there was a dramatic lost in antigen recognition that mirrored the antibody neutralization titers in the 0∆NLS immunized mice. Surprisingly, there was little reactivity to HSV-1 antigen by sera obtained from RING-immunized mice at any time point collected post-boost (Figure 5B). Consistent with previous observations [29], antigen recognition was principally limited to the IgG2b isotype with some recognition by IgG1 (data not shown). Taken together, the results clearly show a significant drop in antibody titers to neutralize and react to HSV-1 over time.

### 2.5. The Immune Cell Profile of Vehicle- and ICP0 Mutant-Immunized Mice Are Similar following HSV-1 Challenge in Long-Term Study

**Figure 6:**
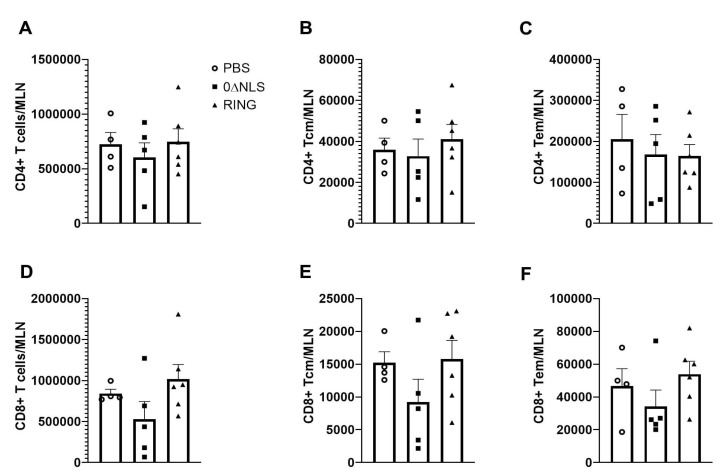
Phenotypic characterization of mandibular lymph node resident cell populations from vaccinated mice post HSV-1 challenge. PBS (vehicle)-, 0∆RING- and 0∆NLS-vaccinated mice ocularly challenged with HSV-1 McKrae (1 × 10^4^ PFU/cornea) 365 days post-boost were exsanguinated at day 7 pi. The draining (mandibular) lymph nodes were removed from the infected mice, processed to single cell suspensions, stained with antibody cocktails and examined for T cell populations by flow cytometry. (**A**) Total CD4^+^ T cells, (**B**) CD4^+^ central memory T cells, (**C**) CD4^+^ effector memory T cells, (**D**) total CD8^+^ T cells, (**E**) CD8^+^ central memory T cells and (**F**) CD8^+^ effector memory T cells are shown with the bars representing the mean + SEM, *n* = 4–6 mice/group. The gating strategy has previously been provided [29].

Previously, we have found a reduction in the infiltration of leukocytes into infected tissue or undergoing clonal expansion in the draining lymph nodes of 0∆NLS vaccinated mice challenged with HSV-1 [26,28,37]. Since there was some degree of protection most pronounced in the 0∆NLS vaccinated group challenged with HSV-1 in the long-term study, we next investigated clonal expansion of lymphocyte populations in vaccinated mice following virus challenge. Analysis of the CD4+ T cell populations found vaccinated mice had similar levels of total CD4+ (Figure 6A), CD4+ central memory (Figure 6B), CD4+ effector memory (Figure 6C), total CD8+ (Figure 6D), CD8+ central memory (Figure 6E) and CD8+ effector memory (Figure 6F) T cells in the MLN 7 days pi. In a similar fashion, HSV-1-specific CD8+ T cells were also found to be similar in frequency and number amongst the vaccinated group of mice indicating no loss in clonal expansion regardless of the vaccine given (data not shown).

**Figure 7:**
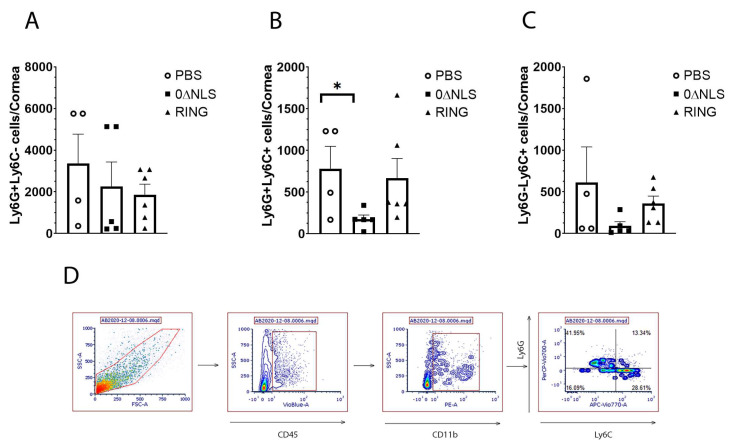
Phenotypic characterization of myeloid cell populations in the cornea of vaccinated mice post HSV-1 challenge. PBS (vehicle)-, 0∆RING- and 0∆NLS-vaccinated mice ocularly challenged with HSV-1 McKrae (1 × 10^4^ PFU/cornea) 365 days post-boost were exsanguinated at day 7 pi. The corneas were removed, processed into single cell suspensions, stained with the antibody cocktail and examined for myeloid populations by flow cytometry. (**A**) Neutrophils (CD45^+^CD11b^+^Ly6G^+^Ly6C^-^), (**B**) inflammatory monocytes (CD45^+^CD11b^+^Ly6G^+^Ly6C^+^) and (**C**) macrophages (CD45^+^CD11b^+^Ly6G^-^Ly6C^+^) are shown with the bars representing the mean + SEM, *n* = 4–6 mice/group. The gating strategy is shown in panel D. * *p* < 0.05 comparing the PBS- to the 0∆NLS-vaccinated groups as determined by ANOVA and Tukey’s post hoc multiple comparison test. (**D**) Gating strategy is shown.

Next, we investigated the infiltration of myeloid cell populations including neutrophils (CD45^+^CD11b^+^Ly6G^+^Ly6C^-^), inflammatory monocytes (CD45^+^CD11b^+^Ly6G^+^Ly6C^+^) and macrophages (CD45^+^CD11b^+^Ly6G^-^Ly6C^+^) into the cornea of vaccinated mice (Figure 7A–D). Whereas neutrophil and macrophage influx into the infected cornea of mice was not altered between vaccinated groups, the number of inflammatory monocytes residing in the infected cornea of 0∆NLS-vaccinated mice was lower than that found in 0∆RING-vaccinated mice and significantly lower compared to PBS-vaccinated animals (Figure 7A–C). CD4+ and CD8+ T cell populations did not average above 100 cells/cornea and were not found to be different between groups of vaccinated mice (data not shown).

**Figure 8:**
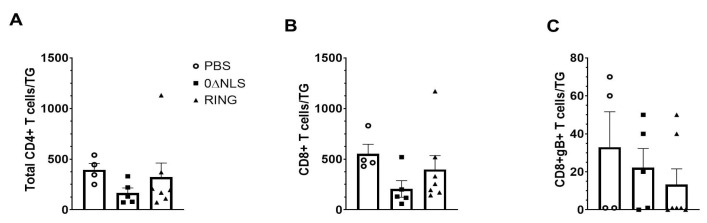
Phenotypic characterization of T cells residing in the trigeminal ganglia (TG) of vaccinated mice post HSV-1 challenge. PBS (vehicle)-, 0∆RING- and 0∆NLS-vaccinated mice ocularly challenged with HSV-1 McKrae (1 × 10^4^ PFU/cornea) 365 days post-boost were exsanguinated at day 7 post infection. The TG were removed, processed into single cell suspensions, stained with the antibody cocktail and examined for T lymphocyte populations by flow cytometry. (**A**) Total CD4^+^ T cells (CD45^+^CD3^+^CD4^+^), (**B**) total CD8^+^ T cells (CD45^+^CD3^+^CD8^+^) and (**C**) HSV-1 glycoprotein B_495–505_ -specific CD8^+^ T cells (CD8^+^gB^+^) are shown with the bars representing the mean ± SEM, *n* = 4–7 mice/group.

Since there was a significant drop in infectious virus recovered from the TG of 0∆NLS- compared to PBS-vaccinated mice (Figure 4E) and CD8+ T cells are instrumental in controlling local infection in the TG [38,39], we next investigated T cell infiltration in the TG post HSV-1 infection in vaccinated mice. The results show no significant differences in the total number of CD4+ (Figure 8A) or CD8+ (Figure 8B) T cells residing in the TG of vaccinated mice following infection. Furthermore, there was no difference in the number of HSV-1 glycoprotein B-specific CD8+ T cells in the TG of vaccinated mice post HSV-1 infection (Figure 8C). Taken together, the drop in HSV-1 titer in the TG of 0∆NLS-vaccinated mice compared to PBS- or 0∆RING-vaccinated animals does not correlate with the number of T cells recruited to the TG during acute infection.

### 2.6. HSV-1 ICP0 Mutant Immunized Mice Are Not Protected from Virus-Induced Corneal Neovascularization upon Challenge One Year Post-Boost

**Figure 9:**
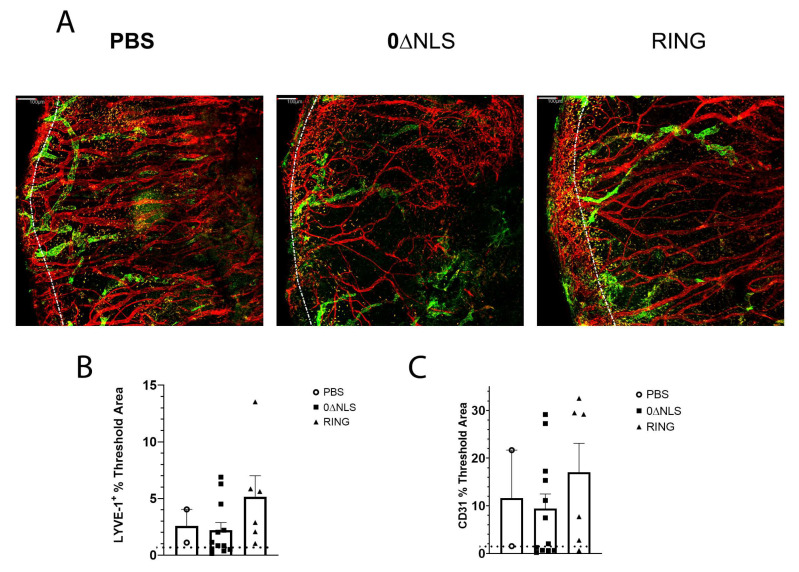
Cornea neovascularization. Mice (*n* = 12/group) were vaccinated with PBS, 0∆RING, or 0∆NLS and subsequently ocularly challenged with HSV-1 McKrae (1 × 10^4^ PFU/cornea). The corneas of mice that survived infection were removed from the eyes of euthanized animals 30 days post infection (pi) and assessed for lymphatic and blood vessel genesis into the central aspect of the cornea. (**A**) Representative confocal images of corneas from vehicle- 0∆NLS- and 0∆RING-vaccinated mice at day 30 pi. Lymphatic vessels appear green and blood vessels appear red. Dotted line outlines the limbus margins. (**B**) Summary of the threshold area of the cornea occupied by Lyve-1+ lymphatic vessels and (**C**) CD31+ blood vessels for each group of mice. Bars represent mean + SEM.

Tissue pathology in the form of blood and lymphatic vessel genesis is observed following acute HSV-1 infection in naive mice [40,41,42,43]. As a means to further compare/contrast long-term vaccine efficacy against acute challenge with ocular HSV-1 infection, assessment of neovascularization (blood and lymphatic vessel genesis) in the cornea was conducted comparing PBS-, 0∆RING- and 0∆NLS-vaccinated mice 30 days pi following infection 365 days post vaccine boost. The results show no differences in the level of new vessel growth comparing the control (PBS)-vaccinated group to either the RING- or 0∆NLS-vaccinated mice 30 days pi (Figure 9A–C). These results are consistent with the results showing similar levels of infectious virus recovered in the cornea of vaccinated mice during acute infection. Thus, unlike what is observed in 0∆NLS-vaccinated animals challenged with HSV-1 30 days post-boost, the efficacy of the 0∆NLS vaccine is lost in terms of preventing corneal neovascularization one-year post-vaccine boost.

### 2.7. Corneal Pathology and Function of ICPO Mutant Vaccinated Mice Are Similar to Control Vaccinated Animals in Long-Term Study

**Figure 10:**
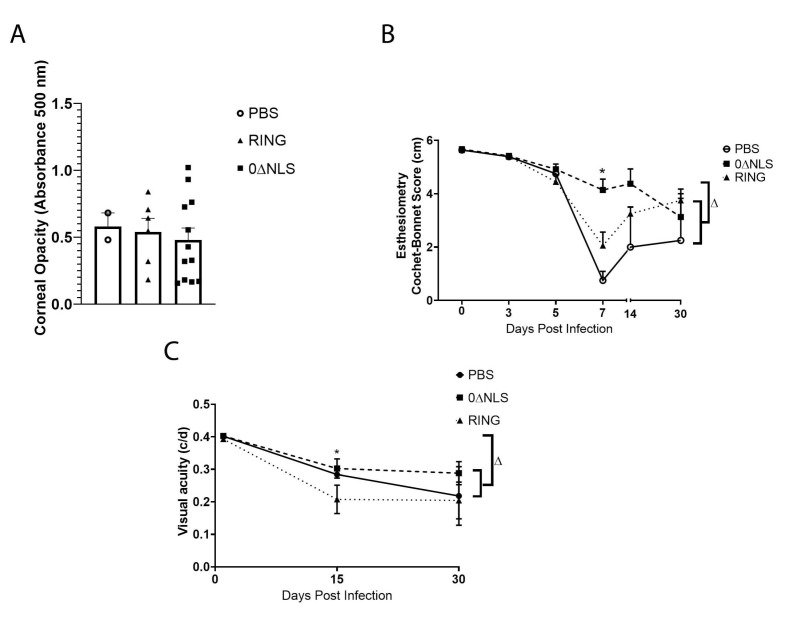
Corneal opacity and function are lost in vaccinated mice challenged one year following vaccine boost. C57BL/6 WT and Rosa male and female mice (*n* = 8–12/group) were vaccinated with HSV-1 0∆NLS (1 × 10^5^ PFU), HSV-1 0∆RING (1 × 10^5^ PFU) or vehicle (PBS) and subsequently ocularly challenged with HSV-1 McKrae (1 × 10^4^ PFU/cornea) 365 days post boost. Mice were assessed for (**A**) corneal opacity at 7 days post infection (pi), (**B**) mechanosensory function and (**C**) visual acuity at the indicated times pi. * *p* < 0.05 comparing the 0∆NLS-vaccinated mice to other vaccinated groups at day 7 pi in (**B**) and comparing 0∆NLS- to 0∆RING-vaccinated mice in (**C**) at day 5 pi as determined by ANOVA and Tukey’s post hoc multiple comparison test. ^∆^ *p* < 0.05 comparing vaccinated groups at day 30 pi to uninfected (day 0 pi) time point as determined by ANOVA and Tukey’s post hoc multiple comparison test in (**C**).

HSV-1 infection of the cornea generates a robust inflammatory response that leads to a loss in corneal innervation [44,45] and typically results in tissue pathology including opacity [46]. Therefore, mechanosensory function that aligns with innervation [47] was evaluated along with cornea opacity in vaccinated mice following ocular HSV-1 infection. In comparison to the control (PBS)-vaccinated animals, 0∆RING- and 0∆NLS-vaccinated mice displayed similar levels of corneal opacity 7 days pi (Figure 10A). However, there were modest differences in mechanosensory function comparing the vaccinated mice temporally following infection. Specifically, there was a pronounced difference between 0∆NLS-vaccinated mice compared to the 0∆RING- or PBS-vaccinated groups at day 7 pi with very little loss in the blink reflex of the 0∆NLS-vaccinated mice compared to the other vaccinated groups that lost between 60–80% of their response (Figure 10B). However, by day 30 pi the blink reflex of the 0∆NLS vaccinated mice had modestly dropped whereas the 0∆RING- and PBS-vaccinated animals blink reflex had partially recovered. Consequently, by day 30 pi there was an overall loss in mechanosensory function that ranged from 30–40% in the 0∆RING- and 0∆NLS-vaccinated mice up to a 63% loss in the control (PBS)-vaccinated group all of which was significant compared to the mechanosensory function of the eye in uninfected, vaccinated mice (Figure 10B).

Visual acuity measurements in free-moving rodents can be accomplished as a behavioral indicator assessed by optokinetic tracking responses [48]. As we previously found a correlation between visual acuity and neovascularization in HSV-1 infected mice [49] and in the current study, neovascularization was found to be similar in vaccinated mice at day 30 pi (Figure 9), visual acuity was determined in vaccinated mice prior to and following HSV-1 infection. Although visual acuity was equivalent among all vaccinated groups prior to infection, mice vaccinated with PBS or 0∆NLS ICP0 mutant had a 25% loss in visual acuity at day 15 pi compared to 0∆RING-vaccinated animals that showed a 50% loss (Figure 10C). By day 30 pi, there was no significant difference between the groups of vaccinated mice but all groups showed a significant loss in visual acuity compared to the corresponding group at the uninfected time point (Figure 10C).

## 3. Discussion

HSV-1 ICP0 mutants are highly immunogenic, live attenuated viruses that were postulated to serve as effective vaccine candidates against HSV-1 infection [50]. In the present investigation, two HSV-1 ICP0 mutants, 0∆NLS and 0∆RING, were evaluated side-by-side in short- and long-term prophylactic efficacy studies against ocular HSV-1 infection. Both ICP0 mutants used as vaccines were found to significantly reduce mortality and virus shedding when immunized mice were challenged 30 days or 1 year following the vaccine booster. Likewise, mice vaccinated with 0∆RING or 0∆NLS mutant virus and infected 30 days post vaccine boost had no detectable infectious HSV-1 in the cornea or TG 7 days pi and this outcome correlated with a lack of tissue pathology in the form of corneal neovascularization and preservation of the visual axis. In contrast to the short-term vaccine efficacy study, only the 0∆NLS-vaccinated mice were found to show a reduction in infectious virus in the nervous system compared to vehicle (PBS)- and 0∆RING-vaccinated animals when challenged 365 days post vaccine boost. Furthermore, 0∆RING- nor 0∆NLS-vaccinated mice retained similar levels of infectious virus recovered from the corneas compared to the control PBS-vaccinated group following virus challenge. Such results show a correlation between cumulative survival and viral load in the central nervous system consistent with other studies that have reported HSV-1 in the nervous system can result in encephalitis and death of the host [51,52]. The lack of efficacy observed in the cornea of ICP0 mutant-vaccinated mice and the resistance in replication and spread in the nervous system in the 0∆NLS-immunized animals in the long-term vaccine study does not seem to have an immune correlate as both antibody neutralization titers and leukocyte infiltration were no different in control and ICP0 mutant vaccinated animals at the time of (in the case of antibody) or post (in the case of leukocyte infiltration) HSV-1 challenge except for a drop in the inflammatory monocyte population recovered in the cornea of 0∆NLS-immunized mice. Since the function of effector T cells was not analyzed in this study, one possibility is that antigen-specific CD8+ T cells that infiltrate the TG and BS of 0∆NLS vaccinated, HSV-1 infected mice may show a greater degree of polyfunctional activity compared to CD8+ T cells from HSV-1 challenged 0∆RING- or PBS-immunized animals as has been noted for 0∆NLS-vaccinated mice infected within 30 days post-vaccine boost [39]. From a humoral standpoint, it is also possible the 0∆NLS-vaccinated animals possess an elevated level of antibody-dependent cell cytotoxicity activity elicited by antigen-driven antibody previously reported to afford protection in unrelated, vaccinated animals from another investigative team [19].

From this study, one can surmise the neutralizing antibody titer does correlate with preservation of the visual axis in that when measurable neutralizing antibody titers were observed in the ICP0 mutant-vaccinated mice, no functional loss or corneal pathology was observed as in the challenge within 30 days post boost. By contrast, ocular tissue pathology in the form of neovascularization and opacity is observed when neutralizing antibody titers are low or non-detectable which is observed in vaccinated mice challenged 1 year following the vaccine booster. The importance of neutralizing antibody in maintaining corneal integrity and function is underscored by passive immunization experiments in which naive mice that receive sera with high anti-HSV-1 neutralizing titers retain a proper functioning visual axis compared to animals that receive sera from naïve animals [26]. CD4+ T cells are required for the ICP0 mutant virus vaccine to generate humoral immunity including neutralizing antibodies as well as generate effector CD8+ T cells that possess polyfunctional activity in response to HSV-1 antigen [27,28]. Whether T cells have a significant impact at the ocular level in direct virus surveillance is not currently known.

The HSV-1 ICP0 mutant viruses are live-attenuated viruses that replicate locally for up to 7 days following footpad administration (Gmyrek and Carr, submitted). Moreover, we have previously shown the 0∆NLS ICP0 mutant virus requires T cell help to elicit a robust antibody response [27] which typically is thought to induce long-lived plasma cells [53]. However, the current results show the antibody neutralization and antigen-recognition titers fade over time to a level similar to vehicle (PBS)-immunized animals. Prior to this study, the HSV-1 ICP0 mutant 0∆NLS has only been evaluated as an effective vaccine out to day 90 post vaccine boost in which it was found to maintain a significant antibody neutralization titer, suppress virus shedding and prevent virus-induced mortality in immunized outbred mice [54]. The long-term study reported herein illustrates the live-attenuated ICP0 mutants do not provide an enduring stimulus to generate a long-lived immune-based memory response that can control virus replication in the cornea and therefore, will likely require additional (periodic) immunizations to maintain a level necessary to preserve cornea integrity and function.

Whereas there was some degree of resistance in 0∆NLS-immunized mice against HSV-1 infection, the protective effect was lost in the cornea when vaccinated mice were challenged one-year post vaccine boost. Not only was there no difference in virus load comparing control (PBS)- to ICP0 mutant-vaccinated groups but the pathology and loss of function of the visual axis was nearly equivalent by day 30 pi. Such results demonstrate the necessity to not only evaluate resistance to infection but also the fidelity of cornea function and visual acuity when assessing the degree of efficacy of a candidate vaccine against ocular pathogens. In fact, these results underscore the significance of interfacing multiple disciplines to ascertain whether an experimental vaccine is relevant to pursue in clinical trials in the case of pathogens that infect tissue-sensitive sites. In summary, we have identified two HSV-1 ICP0 mutants that effectively control ocular HSV-1 infection and limit pathology when used as prophylactic vaccines with a relatively short window of protection as vaccine efficacy wanes by one year after secondary immunization. Whether an additional boost with the ICP0 mutant or a glycoprotein subunit vaccine will retain a strong memory response similar to what was reported for the varicella zoster subunit vaccine [55] has yet to be determined.

## 4. Materials and Methods

### 4.1. Mice

C57BL/6 (wild type, WT) and Ai14/Rosa26-tdTomato Cre-reporter (B6.Cg-Gt(ROSA)26Sortm14(CAG-tdTomato)Hze/J) [56] male and female mice (6–8 weeks old) were obtained from The Jackson Laboratory (Bar Harbor, ME). The animals were kept in a specific pathogen-free vivarium at the Dean A. McGee Eye Institute and University of Oklahoma Health Sciences Center. Mice were anesthetized for all procedures using an intraperitoneal injection of xylazine (6.6 mg/kg) and ketamine (100 mg/kg) and euthanized as described previously [29] or in the case of cumulative survival, monitored out to 30 days pi. Mice were weighed prior to and at days 3, 5 and 7 pi. The percent body weight change (loss) was noted.

### 4.2. Cells, Virus and Virus Growth Curves

Vero cells were obtained from the American Type Culture Collection (Manassas, VA) and used to grow HSV-1 McKrae strain at a stock titer > 1 × 10^8^ PFU/mL. The ICP0-complementing L7 cell line was originally obtained from Neal Deluca (University of Pittsburgh) [57]. Vero cells were propagated in Roswell Park Memorial Institute (RPMI) 1640 medium supplemented with L-glutamine, 10% fetal bovine serum (FBS) and antibiotics (complete media) (ThermoFisher/Life Technologies Ltd., Paisley, United Kingdom) whereas L7 cells were propagated in Dulbecco’s Modified Eagle’s medium (DMEM) supplemented with 10% FBS and antibiotics (DMEM complete media) (ThermoFisher). For growth curve analysis, L7 and Vero cell monolayers were established in 24-well plates at a density of 1.3 × 10^5^ cells/well in 1.0 mL of DMEM or RPMI complete media respectively. One-half of the Vero cell cultures were treated overnight by the addition of 200 units of recombinant IFN-β (RnD Systems, Minneapolis, MN). Sixteen hours later, the cells were infected with 25,000 PFU HSV-1 GFP105, 0∆RING, or 0∆NLS/mL inoculum of each virus [50] to achieve a multiplicity of infection (MOI) of 0.1. Following a 60 min incubation at 37 °C in a 5% CO_2_, 95% air atmosphere to allow for absorption, cell monolayers were rinsed with 1.0 mL DMEM or RPMI complete media and 1.0 mL DMEM or RPMI complete media was added. Cultures were incubated at 37 °C in a 5% CO^2^ 95% air atmosphere. At 18, 24, 36, or 48 hr pi, designated cultures including cells were collected and stored at –80 °C. Thawed samples were subsequently assessed for viral titers by plaque assay using L7 cell monolayers in a standard plaque assay [26].

### 4.3. Immunization and Ocular Infection

WT and Rosa mice were immunized with 1 × 10^5^ plaque forming units (PFU) of the live attenuated HSV-1 0∆NLS, HSV-1 0∆RING [34], or vehicle (PBS) in using a prime-boost regimen in the footpad and flank, respectively as previously described [26]. The prime-boost site of vaccination has previously been found to be the most efficacious in a short-term prophylactic vaccination regimen [54]. Following the initial vaccination, animals were boosted 21 days later and subsequently challenged with 1 × 10^4^ PFU HSV-1 McKrae/cornea 30 (short-term) or 365 (long-term) days post-boost as described [29].

### 4.4. Virus Plaque Assay

For tissue samples, the corneas, trigeminal ganglia (TG) and brain stem (BS) of vaccinated mice infected with HSV-1 were removed and homogenized in 500 µL RPMI complete media. The homogenized samples were centrifuged (10,000× *g*, 1 min) and the clarified supernatant was assayed for viral content by standard plaque assay [26]. Tear samples obtained from the cornea of HSV-1-infected, vaccinated mice were collected by cotton-tipped applicator. The swabs were placed in 0.5 mL of RPMI complete media for determination of virus content by standard plaque assay [26].

### 4.5. Anti-HSV-1 IgG2b ELISA

The serum from non-vaccinated and vaccinated mice was obtained as previously described [29]. The sera were evaluated for virus-neutralizing antibody titers as previously described [26]. Anti-HSV-1 IgG2b in sera was determined by ELISA using immobilized HSV-1 virions on EIA 96-well plates (Costar, Cambridge, MA, USA) as previously described [58]. Other IgG isotypes and IgM titers were considerably lower (i.e., < 0.100 nm absorbance at 405 nm) or below the limit of detection (0.050 nm absorbance at 405 nm) and therefore, not reported.

### 4.6. Ocular Pathology

Corneal opacity was quantitatively assessed as previously described [29] with tissue assayed for absorbance at 500 nm using a FLUOstar Omega plate reader (BMG Labtech, Offenburg, Germany) as previously described [59]. Following the measurement of cornea opacity, the corneas were fixed in a 4% solution of paraformaldehyde (Sigma-Aldrich, St. Louis, MO, USA) for 30 min and washed in PBS containing 1% Triton X-100 (Sigma-Aldrich) [29]. The tissue was then blocked overnight in 10% donkey serum (Abcam, Boston, MA, USA) and labeled for blood and lymphatic vessels as previously described [42]. Image acquisition was obtained using an Olympus FV1200 scanning confocal microscope in sequential scanning channel mode (Center Valley, PA, USA). The total area positive for blood and lymphatic vessels per field of view (4 quadrants/cornea) was quantified using Metamorph software (Molecular Devices Inc., San Jose, CA, USA).

### 4.7. Assessment of Visual Axis Function

Corneal sensitivity was determined using a Cochet-Bonnet esthesiometer as previously described [45]. Briefly, at the indicated time pi, non-anesthetized mice were firmly held and the tip of the monofilament from 0.5 to 6.0 mm in 0.5 mm fractions was touched to the surface of the cornea in four quadrants. The length of the monofilament that elicited a blink response was recorded. The lack of a blink reflex that occurred at the monofilament length of 0.5 mm was recorded as 0.

Spatial visual acuity was evaluated using optokinetic tracking responses measured by an optometry apparatus and software (Cerebral Mechanics Inc., Medicine Hat, AB, Canada). Untrained and unrestrained rodents are placed on an elevated platform in the center of an enclosed container and presented with a 3-dimensional virtual grating that rotates clockwise and counterclockwise. Spatial frequency thresholds are measured and recorded by increasing the spatial frequency until the mice can no longer track the grating in both directions as described [60].

### 4.8. Flow Cytometry

The corneas of infected mice were collected from exsanguinated mice at day 7 pi challenged 365 days post-boost and digested using Liberase TL (Roche, Mannheim, Germany) for 45 min at 37 °C. The processed tissue was filtered through a 40-µm mesh cell strainer (Midsci, Valley Park, MO, USA). The filters were washed with PBS containing 2% FBS. Single-cell suspensions were incubated with anti-CD16/32 (eBioscience, San Diego, CA, USA), labeled with a combination of 1 µL each of CD45 Pacific Blue, CD11b PE, Ly6G PerCP-Cy5.5 and Ly6C APC-Cy7 (Biolegend, San Diego, CA, USA) diluted in 100 µL 1% BSA in 1X PBS for 30 min on ice in the dark. Cells were then washed twice by adding 1 mL of 2% FBS in 1X PBS, centrifuging for 5 min at 300× *g* and decanting the supernatant. Cells were then fixed in 1 mL of 1% paraformaldehyde overnight and resuspended in 1 mL of 2% FBS in 1X PBS to be analyzed on a MacsQuant 196 flow cytometer (Miltenyi Biotech, Bergisch Gladbach, Germany). Samples were analyzed using FlowJo software (Ashland, OR, USA).

Mandibular (draining) lymph nodes (MLN) of vaccinated mice were collected from exsanguinated mice at day 7 pi challenged 365 days post-boost and single cell suspensions were generated by macerating tissue through a 40 µm filter. The staining, gating strategy and analysis was conducted as described previously [29].

Similar with corneas and MLN samples, TG were collected from vaccinated mice exsanguinated 7 days pi challenged 365 days post-boost. Single cell suspensions were generated using a Wheatley Dounce homogenizer (Fisher Scientific, Waltham, MA, USA) in 1 mL of RPMI complete media. Cell suspensions were filtered through a 40 µm filter and washed with 2 mL of RPMI complete media. Single-cell suspensions were incubated with anti-CD16/32 (eBioscience, San Diego, CA, USA), labeled with a combination of 1 µL each of CD45-efluor450, CD3 PECy7 and CD4 APC Cy7 or CD45-efluor450, CD3 PECy7, CD8 APCCy7 and gB-PE diluted in 100 µL 1% BSA in 1X PBS for 30 min on ice in the dark. Cells were then washed twice by adding 1 mL of 2% FBS in 1X PBS, centrifuging for 5 min at 300× *g* and decanting the supernatant. Cells were then fixed in 1 mL of 1% paraformaldehyde overnight and resuspended in 1 mL of 2% FBS in 1X PBS to be analyzed on a MacsQuant 196 flow cytometer (Miltenyi Biotech). Samples were analyzed using FlowJo software (Ashland, OR, USA). All reagents were from eBioscience except the gB-PE tetramer which was obtained from the National Institutes of Health/National Institute of Allergy and Infectious Disease tetramer core facility (Atlanta, GA, USA).

### 4.9. Statistics

Statistical analysis of data was performed using Prism 8 software (version 8.0; GraphPad Software, La Jolla, CA, USA). Data were analyzed between groups using the indicated analysis for statistical significance, *p*-value < 0.05.

## 5. Conclusions

We have identified a second HSV-1 ICP0 mutant attenuated virus, 0∆RING, similar to 0∆NLS, that displays significant efficacy against ocular HSV-1 infection as a prophylactic vaccine in short-term challenge studies in mice. Neither 0∆RING nor 0∆NLS protected the visual axis in terms of performance or function when mice were challenged one- year post vaccine boost. We conclude additional boosts may be required intermittently in order to maintain sufficient resistance to virus-induced pathology of the visual axis.

## Data Availability

The data that support the findings of this study are available from the corresponding author upon reasonable request.
